# The impact of relative energy prices on industrial energy consumption in China: a consideration of inflation costs

**DOI:** 10.1186/s40064-016-2661-z

**Published:** 2016-07-07

**Authors:** Lingyun He, Zhihua Ding, Fang Yin, Meng Wu

**Affiliations:** China University of Mining and Technology, No. 1 Daxue Road, Quanshan District, Xuzhou, 221116 China

**Keywords:** Energy intensity, Energy price, Inflation, Industrial energy consumption

## Abstract

Significant effort has been exerted on the study of economic variables such as absolute energy prices to understand energy consumption and economic growth. However, this approach ignores general inflation effects, whereby the prices of baskets of goods may rise or fall at different rates from those of energy prices. Thus, it may be the relative energy price, not the absolute energy price, that has most important effects on energy consumption. To test this hypothesis, we introduce a new explanatory variable, the domestic relative energy price, which we define as “the ratio of domestic energy prices to the general price level of an economy,” and we test the explanatory power of this new variable. Thus, this paper explores the relationship between relative energy prices and energy consumption in China from the perspective of inflation costs over the period from 1988 to 2012. The direct, regulatory and time-varying effects are captured using methods such as ridge regression and the state-space model. The direct impacts of relative energy prices on total energy consumption and intensity are −0.337 and −0.250, respectively; the effects of comprehensive regulation on energy consumption through the economic structure and the energy structure are −0.144 and −0.148, respectively; and the depressing and upward effects of rising and falling energy prices on energy consumption are 0.3520 and 0.3564, respectively. When economic growth and the energy price level were stable, inflation persisted; thus, rising energy prices benefitted both the economy and the environment. Our analysis is important for policy makers to establish effective energy-pricing policies that ensure both energy conservation and the stability of the pricing system.

## Background

Numerous authors contend that energy (Ayres and Warr 2010; Kümmel et al. 2010) is a driving force of economies. In China, economic revival and development, industrialization and urbanization have depended on the availability of energy resources. In 2011, China passed the U.S. as the world’s largest energy consuming country. Additionally, China is now the world’s largest emitter of sulfur dioxide and greenhouse gases. Thus, energy conservation is now a vital problem for China to solve in its quest to achieve sustainable development. In 2005, China targeted a 20 % energy savings target in its Eleventh “Five-Year Plan.” At the Copenhagen conference in 2009, China promised that its “carbon emissions would decrease by 40–45 % per unit of GDP by 2020.” In fact, over the past decade, China has introduced a substantial number of polices on energy saving and emission reduction, such as the “differential power price policies (No. NDRC^1^ [2005]2254),” the “Adjustment of value added tax policies (No. NDRC [2008]156),” and the “Energy saving and emission reduction action plan for low carbon development in 2014–2015 (No. NDRC [2013]30).” Moreover, China’s Twelfth “Five-Year Plan” for energy saving in 2012 clearly established a target of 21 % for industrial energy consumption during the period of the Plan. The major directives in the Plan indicate first that current energy conservation and emissions reduction efforts should continue. Second, the price reforms of resource-intensive products should be promoted, and the establishment of a price formation mechanism that fully reflects market supply and demand, resource scarcity and the costs of environmental damage should be accelerated. Therefore, the need to reduce carbon emissions has become a significant constraint on China’s economic development process.

The main source of global carbon emissions is energy consumption (IPCC 2014), and China is the world’s largest energy consumer. Thus, the core issue in reducing carbon emissions in China is to reduce energy consumption. In general, given that the current industry-dependent growth model ignores the development of sustainable energy and less carbon-intensive fuels in favor of an exclusive focus on the conservation of traditional energy, there are two main ways to achieve industrial energy savings: controlling the industrial growth rate and reducing energy intensity (Energy/GDP). On the one hand, because China is a developing country and because output is a component of the macroeconomy, its growth rate has not been controlled; on the other hand, energy intensity reflects the issue of energy efficiency, and from a production perspective, there are many factors that affect energy intensity, such as technology innovation (Birol and Keppler 2000; Voigt et al. 2014), the industrial structure (He and Lin 2011; Mi et al. 2014), and the energy structure (Zhu et al. 2015). In practice, technical progress and optimization of the industrial and energy structure cannot be achieved without an effective driving force, i.e., such a change requires some other means to guide and coordinate it. Among these other means, pricing plays a prominent role. The effect of energy prices on the technology, industrial and energy structures has been confirmed by many studies, such as Finn (2000), Birol and Keppler (2000), Wing (2008), and Valadkhani and Babacan (2014). However, China’s energy prices have been controlled by the government for many years and may not effectively reflect supply and demand in the energy market, which means that pricing may not (cannot) play its typical role in which it allocates resources. Reform of the energy-pricing mechanism is thus a central issue in China’s industrial energy conservation efforts (Lin et al. 2007; Wang 2011). Indeed, China’s market-oriented reform of the energy pricing mechanism has achieved great progress.^2^ And Weng’s (2012) empirical research shows that although there is no long-term equilibrium among the main energy varieties, a certain correlation among them has gradually emerged, which is the effect of the energy pricing mechanism reform.

Some of the previous literature has focused directly on the relationship between absolute energy prices and energy consumption, which can reveal a visible relationship between energy prices and consumption. Many studies support the notion that rising energy prices lead to reduced energy consumption (Amano 1990; Martinsen et al. 2007; IMF 2013; Fei and Rasiah 2014; Li and Lin 2015). Some of these studies focus primarily on the channels through which energy prices influence energy consumption. Zhang et al. (2014a, b) find that the stifling effect of rising energy prices on energy consumption is felt most in the transportation sector. Zafeiriou et al. (2014) consider that rising energy prices stimulate consumers’ preferences for new energy sources and eventually lead to reduced consumption of traditional energy. However, Steinbuks and Neuhoff (2014) argue that improvements in energy efficiency and reductions in energy input resulting from rising energy prices are the main reasons for reduced energy consumption. In practice, the own-price elasticity of energy in different industries (He et al. 2014), the purposes of energy consumption (Zheng and Wei 2014), and the sensitivity of energy prices in different areas (Moshiri 2015) all vary.

Most of the research in this field begins by providing a general sense of the pricing mechanisms and analyzes the effects of energy prices on energy efficiency and intensity. Taking a theoretical or empirical approach, Martinez and Ines (2011) find that energy prices are not a key factor in improving energy efficiency, whereas most studies generally confirm a positive relationship between energy prices and energy efficiency, in addition to confirming the positive effects of rising energy prices on industrial energy savings (Birol and Keppler 2000; Fisher-Vanden and Jefferson 2004; Wing 2008; Chen and Wu 2011; Apeaning and Thollander 2013), although a rebound effect also cannot be denied (Brookes 1990; Zha and Zhou 2010; Lin and Liu 2015). Some studies examine the variability that characterizes the relationship between energy prices and energy efficiency or intensity, such as non-linear effects (Kaufman 2004), asymmetric effects (Hang and Tu 2007), dynamic effects (Adofo et al. 2013) and even regional differences (Yang 2011).

Based on the studies described above, it is evident that higher energy prices have an energy saving effect, whereas from a macro perspective, energy prices also have a vital impact on other aspects of the economy. One important aspect is the influence of GDP, which is the focus of many studies. Bashmakov (2007) and Aucott and Hall (2014) examine the percentage of energy costs versus GDP and find that when energy costs increase to over 10–12 %, GDP growth declines, and when energy costs are 5–6 %, GDP growth increases. Another issue that this paper emphasizes is the influence of energy prices on the general price level. Although some studies show that there is no correlation between energy prices and the general price level (Bohi 1991; Jin et al. 2009), a majority of studies generally confirm that there is a positive relationship between energy prices and the general price level (Parks 1978; Thoresen 1983; Cunado and Perez de Gracia 2005; Cologni and Manera 2008; Irz et al. 2013). Some studies measure the conductive influence of energy prices on the general price level (Baffes 2007; Chen 2009), whereas other studies indicate that the relationship between energy prices and inflation has varied over different time periods (Hooker 2002). These studies support the premise of this study that the effects of relative energy prices can be studied from the perspective of inflation costs.

In summary, there is a consensus that increasing energy prices is an effective policy tool for reducing energy consumption. However, most previous studies only examine the direct effects of absolute energy prices on energy consumption. Therefore, we propose the concept of domestic relative energy prices (hereafter to be referred to as relative energy prices), which considers the inflation cost that results from rising energy prices, and we then test the direct, indirect (regulatory) and time-varying effects of relative energy prices on energy consumption. The essence of relative energy prices in this paper is the ratio of energy prices to the general price level of the economy. Moreover, because China is an industrial user of energy (i.e., industrial energy use is greater than domestic energy use, and industrial energy has a greater impact on the level of production in the economy), it may be more appropriate to define the ratio as that of the industrial price index to the overall price index. However, data availability is difficult for industrial energy prices, so to test our concept of relative energy prices, we use domestic prices. Future research could focus on constructing industrial energy price indices so as to improve the explanatory power of this new variable. The significance of this paper is twofold. First, through empirical research, we make clear the extent to which relative energy prices influence energy consumption over our research period. We attempt to determine whether an increasing relative energy price is beneficial to energy saving and whether the effect increases with improvements in the degree of energy pricing that is determined by the market. The results may be particularly instructive for practice. Second, although removing regulations on energy prices is probably good for energy saving in short term, it may result in inflation in the long run. In a market with a higher degree of market pricing, the market itself can guide the energy saving behaviors of the public through the pricing mechanism; on the other hand, the government can regulate energy prices through finance and taxation policies in the long term, which is not only beneficial to energy saving but can also help to avoid inflation.

Following this introduction, the second section of this paper describes the particular pricing mechanism that is operative in China’s energy market. The third section presents an analysis of basic economic theory focused on inflation costs, analyzes the theoretical relationship between energy prices and energy consumption, and then defines relative energy prices, namely, domestic relative energy prices, as the ratio of domestic energy prices (hereinafter to be referred as energy prices) to the general price level of the economy. Additionally, we propose corresponding models. In the fourth section, we empirically measure the direct, regulatory and time-varying effects of relative energy prices on energy consumption. In the fifth section, we examine the asymmetric effects of rising and falling energy prices on energy consumption. The sixth section concludes.

## The pricing mechanism in China’s energy market

**Fig. 1:**
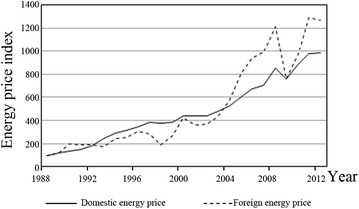
Variations in energy prices in China and foreign countries, 1988–2012. Energy prices in China are measured by indices of raw materials and the fuel purchasing price (1988 = 100); these data are taken from the “China Statistical Yearbook, 2013.” Foreign energy prices are measured by the FOB spot price of Brent crude oil; the data source is the “BP Statistical Review of World Energy, 2013.”

The relationship between supply and demand in a market for energy is embodied in price; therefore, pricing is the core mechanism of resource allocation. Market prices for energy play an important role in guiding industry behavior, including industrial energy consumption. However, a non-market price will weaken the resource allocation effects of pricing. Regarding China’s energy-pricing mechanism, energy prices in the country were completely controlled by the government prior to 1978. After 1978, China implemented its reform and opening-up policies, and the government relaxed its intervention in the commodities market. However, because energy is a basic commodity that greatly affects social production and life, the government maintained absolute control in its pricing and management. In 1992, the government clearly enunciated a strategic goal of establishing a socialist market economic system, and market pricing mechanisms have been gradually introduced to energy industries since that time. The gradual expansion of the volume of market adjustments has led to less control over prices, and in particular, the price of energy has been allowed more freedom. Overall, due to the reform and opening-up policies, some features of China’s energy-pricing mechanism were shown to be hierarchical, multi-stepped and incremental, which led to continuously rising energy prices. However, energy prices in China have not been fully liberalized, and they remain obviously lower than foreign energy prices. Figure 1 compares energy prices in China with those of foreign countries^3^ and shows that on the one hand, energy prices in China are obviously lower than foreign prices overall and that on the other hand, changes in foreign energy prices are more flexible and more volatile.

**Fig. 2:**
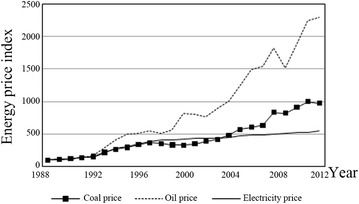
Variations in the main types of energy prices in China. The related data are the fixed base data (1978 = 100), and the data source is the “China Statistical Yearbook, 2013.” These data are not needed for the empirical analysis because we only want to capture the movement trends of different energy prices (we do not change it into data 1988 = 100)

Regarding the main types of energy, prices rose during the sample period, and variations in the coal and oil prices are obvious, whereas the price of electricity rose only modestly (Fig. 2). The Chinese government decided to liberalize the pricing mechanism for coal in 1993; in 2002, it announced that the guiding price of coal would not be published, and the market-oriented pricing of the coal industry would essentially be realized. From 2002 to 2012, rapid growth in the Chinese economy brought enormous demand for coal, and coal prices therefore increased rapidly. This period is known as the “golden ten years” of the coal industry’s development. With respect to oil, the government managed and monopolized its pricing until 1998. After 1998, the pricing mechanism gradually changed from government pricing to guidance pricing, and the price of oil in China began to move with prices on international markets. After 2001, the government took the refined oil pricing mechanism reform further, and the price of oil tended to be decided by both the government and the market, particularly after the reform of 2009. As for the price of electricity, a market completion mechanism was introduced to the power generation sector in 1985, and the government undertook separate but related reforms in 2002 and 2003. However, these reforms only served to separate power plants from the grid, and the monopoly remained unbroken. Overall, since 2002, progress has remained slow in electricity pricing reform. Although China has established a competitive electricity market, substantial government restrictions remain.

Based on this background, this paper attempts to analyze the impact of energy prices on energy consumption in China and then objectively locates the function of energy prices in the field of energy savings and emissions reduction.

## Theoretical framework and model construction

### The definitions of relative energy prices and energy consumption

The essence of relative energy prices is the relationship among prices. Taking the definition of relative energy prices at home and abroad (Wei and Lin 2007; Yang 2009)^4^ as a reference, we consider the relative energy price as a comparison between energy prices and the general price level from the perspective of inflation costs. Thus, we do not consider relative prices based on other factor inputs such as capital and labor or through price comparisons among different types of energy (Wing 2008). Moreover, regarding energy consumption, there are also two levels of analysis: total energy consumption and energy consumption intensity.

Based on the above analysis, we first affirm the linkage between energy prices and the general price level. According to Lin and Wang (2009), the range of price variations can be expressed as

1 $$\Delta P_{j} = \sum\limits_{i = 1}^{n} {m_{ij} \Delta P_{i} } ,\quad {\text{j}} = 1,2, \ldots ,{\text{n}},$$

where ∆*P*_*i*_ and ∆*P*_*j*_ are the price variations of commodity *i* and *j*, respectively; the price variation of commodity *j* is influenced by the price variations of all the commodities related to *j*; m_*ij*_ is the direct consumption coefficient of commodity *I*; and *j* is an input–output table. This equation can be rewritten as

2 $$\Delta P_{n - 1,1} = \sum\limits_{n = 1}^{N} {m_{n,n - 1} \Delta P_{n,1} }$$

where ∆*P*_n_ is the range of variation in energy prices. As for the original order n in the direct consumption coefficient matrix M in the input–output table, transposing the order n − 1 in the direct consumption coefficient matrix after removing row n and column n, we have

3 $$\Delta P_{n - 1,1} = \sum\limits_{n = 1}^{N} {\mathop {\left[ {(E - M_{n - 1} )^{ - 1} } \right]^{T} m}\nolimits_{n,n - 1} } \Delta P_{n,1}$$

where *E* is an n × n identity matrix.

To obtain the general price level, a price index $\overline{\Delta P}$ is generally constructed as a weighted average of the prices of related commodities. Thus, the impact of energy prices on the general price level can be expressed as

4 $$\overline{\Delta P} = \sum\limits_{n = 1}^{N} {\mathop \theta \nolimits_{n - 1,1} } \left( {\sum\limits_{n = 1}^{N} {\mathop {\left[ {(E - M_{n - 1} )^{ - 1} } \right]^{T} m}\nolimits_{n,n - 1} } \Delta P_{n,1} } \right)$$

where *θ*_*n*−1_ is the proportion of commodity *n* − 1 in the price index compilation.

This analysis shows that rising energy prices inevitably lead to increases in other related product prices and finally increase the possibility of inflation. Inflation can be regarded as a type of policy cost because it deviates from the target of macro-regulation and will increase the costs of the central bank’s reaction to inflation (Fiore et al. 2006). In addition, both unexpected as well as expected inflation generates costs for society (Han 2004), including menu costs, shoe-leather costs and welfare costs, among others (Chiu and Molico 2010; Lee 2013; Nakata 2014). In fact, almost any cost is the definite result of a constantly increasing general price level that may lead to low efficiency in the economy in general (Heer and Sussmuth 2007; Bick 2010; Schneider 2014). Therefore, in this paper, we do not focus on the inflation cost itself but consider inflation (a constant increase of the general price level) as a comprehensive policy cost, which means that achieving energy savings by increasing energy prices is bound to create an inflation cost in practice.

Based on the foregoing, we define $relative\;energy\;prices = \frac{energy\;prices}{general\;price\;level}$; here both the relative energy prices and energy prices are domestic indices. Thus, we can use research on the effects of monetary policy as references. Generally speaking, the basic coefficient is BCE = GDP growth indexation/CPI, where GDP growth indexation is profit and CPI is the cost of monetary policy. Using this equation as a reference, we consider that energy prices can impact both energy consumption and the general price level. The former represents profit, while the latter represents the cost of the pricing policy. Then, the ratio of the two components is the effect of the energy price lever, which includes consideration of energy conservation. In addition, Zhang et al. (2014a, b) show that once inflation is considered, a central bank’s monetary policy that aims to control inflation may also influence energy prices, which means that there is a transmission chain through “energy prices–price level–energy prices” in practice. Moreover, excluding the general price level in examining energy prices not only may reflect the real variation in energy prices but also may consider the cost of price lever regulation and even the endurance of the economy’s price system, which is more instructive in practice. It is through this mechanism that Amano (1990) indicates that rising real energy prices may result in energy savings. In general, the inflation cost in this paper fundamentally includes two aspects: first, according to the analysis described above, inflation itself is a cost of pricing policy; second, inflation may result in many costs, such as shoe-leather costs, menu costs and tax distortions. In this paper, we do not focus on the specific costs in detail. We only assume that all costs are reflected by the general price level. Then, the question becomes, how can the general price level be excluded in examining energy prices? Using the relationship between nominal variables and real variables (such as the nominal and real interest rate, the nominal and real product price index, and nominal and real energy prices) as a reference (Yamada 2002; Shen and Wang 2000; Yang 2009),^5^ we first consider the difference between energy prices and the general price level. However, the difference between the two indices may be negative, which prevents the use of the natural logarithm and influences the models in empirical research, and the absolute value of the difference does not reflect rising and falling price variations. Therefore, the ratio between the two indices can reasonably be adopted. Based on the foregoing, we utilize relative prices to reflect the real energy prices such that the inflation cost of energy price variations is excluded, and the effects of relative energy prices on energy consumption are therefore analyzed.

### Theoretical relationship between energy price and energy consumption

According to general commodities theory, the relationship between energy prices and energy consumption essentially belongs to the “price-demand” research framework. This relationship can be interpreted based on two components, the factors involved and market equilibrium. As for the factors involved, energy prices are important factors in production, and an increasing energy price can thus result in increasing costs for related products, whereas a high energy consumption industry may accelerate its industrial transition and ultimately lead to decreasing energy consumption. Furthermore, to operate continuously and maintain a profit margin and a sustainable competitive advantage, enterprises may increase their technology input and actively search for alternative energy sources. As for market equilibrium, energy demand in the industrialization process is rigid, and energy supply and demand is imbalanced. Thus, rising energy prices can stimulate energy enterprises to expand the scale of their production and sales, which is bad for energy conservation.

In the real economy, energy is both a factor input and also is general merchandise. Energy prices can regulate energy consumption by influencing the economy in the aggregate, the economic structure, and energy efficiency. A correlation between energy prices and the economy in the aggregate has been confirmed by many scholars, although whether it is positive or negative is inconsistent and depends on different economic conditions (Jin et al. 2009; Berk and Yetkiner 2014; Bretschger 2015). As for the path of the economic structure, Hu et al. (2008) indicate that energy prices not only can lead to changes in the economic structure but also can influence the internal industrial structure. This influence can be confirmed through the theory of sector transfer. As for energy prices and energy efficiency, rising energy prices generally stimulate technological innovation and decrease energy consumption, which was summarized in the introduction of this paper. Finally, it must be stressed that because energy intensity is the ratio of total energy consumption to GDP—and because energy prices can impact both of these components (at least theoretically)—energy prices may certainly influence energy intensity.

### The basic model

#### The direct effects of relative energy prices on energy consumption

Many factors affect energy consumption. Existing studies mainly conclude that factors influencing energy consumption include both productive factors, such as output, the industrial structure, and technological efficiency, and consumptive factors, such as population, and per capita wealth. Based on the Kaya equation and LMDI decomposition (Ang and Liu  2007; Ma and Stern 2008; Ang 2015), we have:^6^

5 $$CO_{2} \,emissions = C_{e} \times E_{i} \times Y_{p} \times P_{o}$$

where *C*_*e*_, *E*_*i*_, *Y*_*p*_ and *P*_*o*_ are the carbon content of the energy, energy intensity, GDP per capita and population, respectively. This paper mainly focuses on the productive factors, and then we consider the carbon content of energy, energy intensity and GDP. 7 In addition, studies of factors affecting the carbon content of energy, energy intensity and GDP (we cited the related studies in the background section) mainly focus on technology innovation and the industrial and energy structure; meanwhile, the energy price is taken as a driving factor of technology innovation and the industrial and energy structure. Based on the above analysis, we finally have:

6 $$Energy\,{\kern 1pt} consumption = Y \times E \times S \times T \times P$$

where *Y*, *E*, *S*, *T* and *P* are the aggregate output, the energy structure, the industrial structure, the technological level and energy prices (relative energy prices in this paper). These parameters have been observed to directly affect energy consumption. The direct effect model is shown in Eq. (7). To eliminate heteroskedasticity and directly obtain elasticities, natural logarithms of the variables are used in the model. *C*_1_ and *C*_2_ represent total energy consumption and energy intensity, respectively.

7 $$lnC_{i} = \alpha + \beta_{1} lnY + \beta_{2} lnS + \beta_{3} lnE + \beta_{4} lnT + \beta_{5} lnP$$

From a practical perspective, the most significant flaw of Model (7) is that price is treated as the central mechanism of resource allocation. This model aims to measure the direct influence of the related variables, especially relative energy prices, on energy consumption. Moreover, prices could also affect energy consumption through their influence on variables such as the aggregate output, the industrial structure, and the energy structure. Thus, the key is to highlight the regulatory effects of relative energy prices on energy consumption.

#### The regulatory effects of relative energy prices on energy consumption

Based on the analysis in the section of “Theoretical relationship between energy price and energy consumption” and fully considering the regulation of relative energy prices on energy consumption, the model might be rewritten as (8):

8 $$\begin{aligned} lnCi &= \alpha + \beta 1ln(P \times Y) + \beta 2ln(P \times S) + \beta 3ln(P \times E) \\ &\quad + \beta 4lnT + \beta 5lnP + \beta 6lnY + \beta 7lnS + \beta 8lnE \end{aligned}$$

In Eq. (8), the relative price of energy might influence energy consumption by regulating related variables. The cross-product items represent the regulatory process. They aim to measure the indirect effect of relative energy prices on energy consumption through the influences of intermediary variables such as aggregate output, the industrial structure, and the energy structure. Obviously, there is significant multicollinearity in this model under the general regression method. However, in view of their economic significance, the above variables must be included in the model. Thus, the ridge regression method is utilized to analyze the actual operations.

#### The time-varying effects of relative energy prices on energy consumption

Models (7) and (8) capture the effects of energy prices on average energy consumption. However, the influence of energy prices on energy consumption exhibits a remarkable time-varying characteristic with variations in the macroeconomic environment and policy conditions. Thus, a state-space model (SSM) may better reflect the dynamic nature of these relationships. It should be noted, however, that SSMs have difficulties with multicollinearity. Therefore, the regulating variables are not included in Model (9). Among the variables included in the empirical study, some must be removed based on causality tests. The model aims to capture the dynamic effect (time-varying coefficients) of relative energy prices on energy consumption.

9 $$\begin{aligned} lnC_{it} & = C + \beta_{1,t} lnY_{t} + \beta_{2,t} lnS_{t} + \beta_{3,t} lnE_{t} + \beta_{4,t} lnT_{t} + \beta_{5,t} lnP_{t} \mu_{t} \\ \beta_{1,t} & = C(1) \times \beta_{1,t - 1} + \varepsilon_{1,t} ,\beta_{2,t} = C\left( 2 \right) \times \beta_{2,t - 1} + \varepsilon_{2,t} \\ \beta_{3,t} & = C(3) \times \beta_{3,t - 1} + \varepsilon_{3t} ,\beta_{4,t} = C\left( 4 \right) \times \beta_{4,t - 1} + \varepsilon_{4t} \\ \beta_{5,t} & = C(3) \times \beta_{5,t - 1} + \varepsilon_{5t} \\ \end{aligned}$$

## Measuring the effects of relative energy prices on energy consumption

### Sample selection and pretreatment

**Fig. 3:**
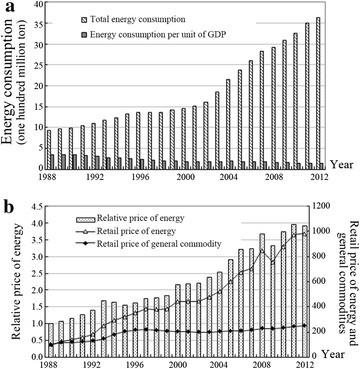
Variations in energy prices and energy consumption in China, 1988–2012. In this figure, energy prices in China are measured using indices of raw materials and the fuel purchasing price (1988 = 100), and the general price level is measured by the retail price of commodities (1988 = 100). The definition of the relative energy price in this paper is the ratio between the two components. The related data are taken from the “China Statistical Yearbook, 2013.” **a** Energy consumption (total amount and intensity); **b** energy prices (absolute and relative)

The relative energy price has been replaced by the ratio of the index of raw materials and fuel purchasing price to the retail price of commodities (RPI).^8^ The GDP index with a base year of 1978 was selected as the total output variable. The GDP ratio (%) of the second industry was treated as the industrial structure, and the proportion of coal (%) in energy consumption was selected as the energy structure. RandD input was treated as the technology level. The sample period was 1988–2012. The data^9^ show variations in energy prices and in energy consumption during the sample period (Fig. 3a, b).

During the sample period, the price of energy in China exceeded the general price level of retail commodities and exhibited greater fluctuations. Relative energy prices tended to increase gradually, which shows that energy prices came to better reflect fundamentals. Concretely speaking, because of the special administrative pricing mechanism in the Chinese energy market, energy prices were lower than real market prices over the long term and did not reflect supply and demand on the market. Increases in energy prices indicated that the price had become more rational. Beginning in 2002, total energy consumption in China increased significantly, which conforms to the expansionary nature of the Chinese economy. Moreover, because China’s economic development is essentially dependent on industrial production, total energy consumption was difficult to control. However, based on energy intensity, it is evident that energy consumption per unit of GDP gradually decreased year by year. There are two main reasons for this development. First, rapid economic growth induced increased GDP cardinality. Second, restraints on carbon emissions continued to increase, gradually shifting attention to improved energy efficiency in China. Overall, whereas variations in relative energy prices corresponded to variations in total industrial energy consumption, the opposite was the case for energy intensity. However, in some cases, relative energy prices significantly increased during periods of relatively stable energy consumption. Thus, the actual relationship must be explored further.

Following the requirements of the model, a stability test was performed in the appropriate sequence. The results show that all sequences were I (1). A further cointegration test was conducted on this basis (Table 1).

**Table 1:** Cointegration test results of relevant variables and energy consumption

Energy consumption	Null hypothesis	Characteristics of the root	The trace statistic	5 % critical value	P value
C_1_	None^a^	0.8654	103.8884	69.8189	0.0000
	At most 1^a^	0.7213	57.7710	47.8561	0.0045
	At most 2	0.5630	28.3861	29.7971	0.0721
C_2_	None^a^	0.8910	115.4177	69.8189	0.0000
	At most 1^a^	0.7965	64.4490	47.8561	0.0007
	At most 2	0.5816	27.8306	29.7971	0.0829

^a^Rejection of the null hypothesis at the 5 % confidence level

The test revealed a relationship of cointegration between the sequences of lnC_i_ and lnY, lnS, lnE, lnT and lnP. The result indicates that the factor variables and energy consumption have an equilibrium relationship over the long term. On this basis, a Granger causality test was performed on the sequences of C_i_ and the relevant influencing factors. To preserve as much information from the original sequence as possible, the test was first conducted based on the original sequence. For these sequences, causal relationships were determined by whether they passed through the test, or the test would be carried out after the difference. The test results are shown in Table 2.

**Table 2:** The Granger causality test for relevant variables

Null hypothesis	Statistics^†^	Null hypothesis	Statistics^†^	Null hypothesis	Statistics^†^
Y → C_1_	2.0037 (2) [0.1638]	ΔY→ΔC_1_	2.8246 (5) [0.0927***]	Y → C_2_	2.8302 (2) [0.0854***]
S → C_1_	0.8978 (2) [0.4249]	ΔS→ΔC_1_	3.8512 (5) [0.0449**]	S → C_2_	3.5116 (1) [0.0749***]
E → C_1_	4.1082 (2) [0.0339**]	E→C_2_	3.4185 (2) [0.0552***]	T → C_1_	4.0765 (2) [0.0347**]
T → C_2_	2.0846 (2) [0.1534]	ΔT→ΔC_2_	0.5987 (2) [0.5607]	P → C_1_	2.6105 (2) [0.1011]
ΔP → ΔC_1_	0.6759 (2) [0.5218]	P→C_2_	1.8399 (2) [0.1875]	ΔP → ΔC_2_	0.2398 (2) [0.7894]
P → Y	16.9391 (1) [0.0005*]	P→S	4.7373 (2) [0.0222**]	P → E	3.2816 (4) [0.0491**]
P → T	2.4996 (4) [0.0982***]	–	–	–	–

*^,^ **^,^ *** Rejection of the null hypothesis at the 10, 5 and 1 % levels, respectively

^†^Consists of the F statistic (the lag phase) [the P value]

Whereas relative energy prices contribute to variations in total energy consumption in the short term, they play a more significant role in the influence of the industrial structure on energy consumption. At the same time, energy consumption also results in variations in total industrial output, whereas the relationship between total industrial output and total energy consumption must still be determined. Energy intensity and energy structure showed reciprocal causation, which demonstrates that energy structure optimization played a vital role in energy conservation during the sample period. Moreover, the industrial structure has a unidirectional impact on energy intensity, and relative energy prices might influence both the energy structure and the industrial structure. Thus, relative energy prices indirectly regulated energy intensity in China. In addition, a causal relationship is evident between relative energy prices and the technology level. Technological advances and increased technology inputs can result in increased energy prices. Conversely, rising energy prices also contributed to technological development and influenced energy consumption during the sample period.

### Direct effect

The above test shows that relevant sequences can meet the needs of the model test. Accordingly, based on the theoretical analysis, the direct effectiveness of the variables was measured using Model (7). The model continues to be adjusted to achieve optimal results (Table 3).^10^

**Table 3:** The regression results regarding the direct effectiveness of the relevant variables with respect to industrial energy consumption in China

Variables	C_1_		C_2_	
C	–	–	−8.351 (−4.834***)^†^	−12.283 (−6.91***)
Y	−1.148 (−1.780*)	0.892 (11.644***)	−0.870 (−3.856***)	–
S	3.423 (6.083***)	1.649 (11.526***)	−0.128 (−0.410)	−0.857 (−3.18***)
E	–	–	3.058 (9.122***)	3.869 (10.63***)
T	1.045 (3.118***)	–	0.355 (−3.117***)	–
P	−0.761 (−2.523**)	−0.337 (−5.159***)	−0.108 (−0.934)	−0.250 (−6.04***)
	R^2^ = 0.953 DW = 0.582	R^2^ = 0.996 DW = 1.903	R^2^ = 0.990 DW = 1.878	R^2^ = 0.981 DW = 1.969

*^,^ **^,^ *** Rejection of the null hypothesis at the 10, 5 and 1 % confidence levels, respectively. For the regression model C_1_, which includes Y, S and P, the iterative estimation method is used because the regression results that use OLS are not sound

^†^The values in parentheses are t value

We observe the following:

First, the variable T was not found to be significant in the direct effect model. Combining the above analyses, over the sample period, the direct effect of the technology level on energy consumption was found to be insignificant. The main reasons for the increase in total energy consumption in China were increasing output, an increasing industrial share of output and extensive economic growth, whereas the coal-dominated energy consumption structure was the key factor in the rising energy intensity. The elasticity of the industrial structure with respect to total energy consumption and energy intensity was 1.649 and −0.857, respectively. This finding reveals that a decline in the industrial share of output can reduce total energy consumption and result in environmental benefits. However, as a result of its inhibiting effect on production, energy consumption per unit of GDP increased, which indicates that under the current conditions, it is critical to optimize the internal industrial structure instead of reducing the industrial share of output in the national economy.

Second, relative energy prices might impact total energy consumption and intensity, with coefficients of −0.337 and −0.250, respectively, which highlights the important role of rising energy prices in energy conservation. Rising energy prices add to the production costs of enterprises and stimulate energy-intensive enterprises to improve production efficiency through technology or other means. However, because of the administrative energy-pricing mechanism in China, energy prices cannot reflect supply and demand in the energy market, and they also cannot provide the full influence of their fundamental role in resource allocation; thus, the direct effect of relative prices on energy conservation was thus less than that of industrial restructuring. Therefore, both factors should be coordinated in real economic development. However, the inevitable choice of the more in-depth internal marketization reform worked to promote the effectiveness of the regulation of energy prices in influencing industrial energy consumption. It should be noted that in practice, the government always compensates for lost industrial energy consumption to ensure the overall welfare, which can be reflected in the income effect and may reduce the effects of price on energy savings.

### Regulatory effect

In actual market-driven situations, the market performs the function of allocation, and energy prices play a regulatory role in energy consumption through related variables. On this basis, Model (8) can be used to analyze the regulatory effect of relative energy prices on total energy consumption and intensity. Moreover, to solve the problem of multicollinearity in a regulatory effects model, the ridge regression method is used for measurement purposes (Table 4A, B).

**Table 4:** The regulatory effects of relevant variables on (A) total energy consumption (C_1_), (B) energy consumption per unit of GDP (C_2_)

Regulatory items	Variables			
	P × Y	P × S	P × E	P
(A)				
Y	0.266	–	–	0.340
S	–	0.247	–	0.217
Y + S	0.064	0.286	–	−0.097
S + E	–	0.237	0.237	−0.104
Y + S+E	0.060	−0.018	0.353	0.228

Regulatory items	Variables				
	P × Y	P × S	P × E	P	T
(B)					
S	–	−0.183	–	−0.188	−0.606
E	–	–	−0.214	−0.213	−0.536
S + E	–	−0.144	−0.148	−0.143	−0.538
Y + S+E	−0.204	−0.147	−0.148	−0.147	−0.239

Relative energy prices affect total industrial energy consumption and intensity in China. However, depending on the regulation path, the effects vary in the following ways:

1. Given a single regulation path, relative energy prices have a positive and direct effect on total industrial energy consumption in China. This result is inconsistent with theory. Due to the non-market energy-pricing mechanism in China, it was expected that the allocation effect of price on consumers would not be significant in China. Because of low energy prices over the long term, the industrial energy market was unable to reach a balance between supply and demand, with surplus lost on both sides (production and consumption) of the energy market. In addition, relative energy prices might influence total energy consumption via overall economic output and structure, which exhibit regulatory effects of 0.266 and 0.247, respectively. This finding indicates that rising relative energy prices may foster economic growth by way of social consumption, leading to increased energy consumption. Additionally, given the goal of industrialization, rising energy prices might increase production costs in the energy-intensive industrial sector. However, the organization of Chinese enterprises plays a special role. In state-owned and large industrial enterprises, capital and labor inputs increased. At the same time, massive subsidies from the government counteracted the decline in energy consumption through the income effect. Thus, improved industrial production induced high total industrial energy consumption. Therefore, the optimization of the industrial structure and rational energy-pricing policies should be coordinated. Inevitably, it was decided to suppress total energy consumption in China.
2. In the comprehensive regulation path, relative energy prices directly inhibited industrial energy intensity, whereas technological progress directly fostered a decline in energy intensity. Relative energy prices had regulatory effects on energy consumption through the economic structure and the energy structure, where their effects were −0.183 and −0.214, respectively. When both the economic structure and the energy structure are considered, the regulatory effects were −0.144 and −0.148, respectively. These effects demonstrate that higher energy prices constrained the increase in energy intensity through both the industrial structure and the energy structure. Rising relative energy prices induced increased energy consumption costs, accelerated technological innovation and reduced energy consumption.

### Time-varying effect

Based on Model (9), the time-varying parameters in the SSM have been applied for measurement purposes. According to the above results, the correlation between T and P was high, but the SSM could not effectively address the multicollinearity issue. Thus, Y, S, E and P were the only parameters included in the model. The SSM has been established with C_1_ and C_2_ as explanatory variables, and the results are presented in Table 5.

**Table 5:** The results of the state-space model

Energy consumption	State parameter	Final state	Root MSE	z-statistic	Prob.
C_1_	SV_Y	0.800249	0.062752	12.75255	0.0000***
	SV_S	−0.866024	0.219378	−3.947634	0.0001***
	SV_E	3.414087	0.183423	18.61323	0.0000***
	SV_P	0.041590	0.106340	0.391105	0.6957
C_2_	SV_Y	−0.199939	0.062757	−3.185908	0.0014***
	SV_S	−0.866359	0.219397	−3.948824	0.0001***
	SV_E	3.412813	0.183438	18.60470	0.0000***
	SV_P	0.041810	0.106349	0.393139	0.6942

*^,^ **^,^ *** Rejection of the null hypothesis at the 10, 5 and 1 % confidence levels, respectively

**Fig. 4:**
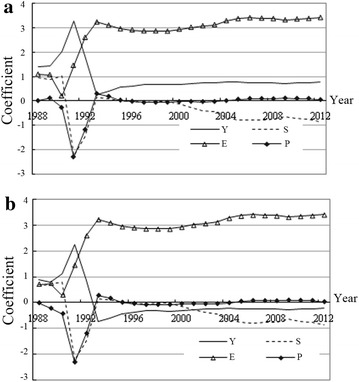
The time-varying trajectories of the effects of the relevant variables on C_1_ and C_2_: **a** C_1_, **b** C_2_

In the above results, a good fit is observed for both models. The time-varying parameters could thus be generated, reflecting variations in the effects of the relevant variables on C_1_ and C_2_ in the sample period (Fig. 4).

Overall, there were minimal differences in the effects of Y, S, E and P on C_1_ and C_2_, including the size, direction and dynamic variation tendency. Moreover, the effect of the related variables on energy consumption seems to be quite volatile in the early years (1989–1992). In fact, China suffered serious inflation in 1988, and the following 3 years were the period of “Reorganization.” It was not until 1992 that the economy recovered to a certain extent. The volatility indicates that the economic environment may greatly influence the relationships among the related variables. In particular, after the 1990s, industrialization and urbanization in China accelerated. The development model followed by China was energy-intensive and characterized by high energy consumption. Accordingly, economic growth led to accelerating energy consumption. Since 2000, the energy-saving effect associated with economic structure optimization has been prominent. This result is consistent with the test results for the two models above: the effective path to declining total energy consumption and intensity involves optimizing the energy consumption structure. Specifically, coal consumption was low before 1999, and both total energy consumption and intensity decreased. From 2000 to 2012, the coal market became prosperous, and the reforms were continued, with the share of coal in energy consumption continuing to increase, which fostered substantially increased energy consumption. With the end of the “ten golden years” of coal, energy restructuring entered a new phase, thus providing a crucial opportunity to reduce industrial energy consumption. In the sample period, the impact of relative energy prices on energy consumption was significantly less than that of other factors. There were also clear period-related differences.

1. From 1993 to 1995, relative energy prices were positively correlated with total energy consumption and intensity. In practice, the price index of retail commodities continued to increase during this period, although energy prices during this time were relatively low, which stimulated the procurement of productive factors, and then energy consumption increased. This finding shows that given low economic development, a high price level and limited endurance of the pricing system, rising energy prices did not help to control energy consumption. Thus, undertaking market-oriented reforms is not conducive under this condition.
2. From 1996 to 2003, relative energy prices were negatively correlated with energy consumption. In this phase, the price index of retail commodities slightly declined, whereas energy prices increased rapidly, and energy scarcity was evident. Rising energy prices resulted in increased production costs, which led to declines in both total energy consumption and energy intensity. Thus, during this period, given that economic growth and the price level were relatively stable, inflation persisted, and rising energy prices benefitted both the economy and the environment. This situation presented a good opportunity for energy market reform.
3. From 2004 to 2012, relative energy prices were positively correlated with energy consumption. In this phase, the price index of retail commodities continued to rise. Inflation pressures increased—particularly after China’s 4 trillion yuan stimulus package in 2008. However, energy prices were also rising rapidly, with a tendency that exhibited complex fluctuations. Thus, given the rapid economic growth, high price levels, and insufficient endurance of the price system, energy-pricing reform was not appropriate under these conditions.

Overall, the market-based energy-pricing mechanism was an effective means of energy conservation. However, reform of energy pricing must be coordinated with macroeconomic and industrial policies. It is also vital to select the right market opportunity; otherwise, such a reform will not only adversely impact economic growth and price stability but also adversely affect environmental efforts.

## Measurement of the asymmetrical effects of relative energy prices on energy consumption: a further study

According to general economic theory, energy prices influence total energy consumption and intensity through the factor substitution effect and the technological progress effect, which was briefly discussed in the third section of this paper. In general, rising energy prices lead to decreasing energy consumption. Conversely, with declining energy prices, energy consumption will increase. However, China’s economy is in the process of rapid development as the material basis for economic growth, energy demand is rigid, and the reaction of energy consumption to changes in energy prices may differ depending on whether energy prices rise or fall. Therefore, the impact of energy prices on energy consumption may be asymmetric. A further examination is presented in the following section.

### Methodology and data used

Based on Model (7), two dummy variables, A_t_ and D_t_, are included in the model to represent the rise and fall of relative energy prices to measure asymmetry. We express Formula (10) as follows:

10 $$A_{t} = \left\{ {\begin{array}{*{20}l} 1 \hfill &\quad {\Delta P_{i} > 0} \hfill \\ 0 \hfill &\quad {\text{Others}} \hfill \\ \end{array} } \right.\quad D_{t} \, = \left\{ {\begin{array}{*{20}l} 1 \hfill &\quad {\Delta P_{i} < 0} \hfill \\ 0 \hfill &\quad {\text{Others}} \hfill \\ \end{array} } \right.$$

Let dummy variables be substituted into Model (5), namely, let $\beta_{5A} (A_{t} lnP_{t} ) + \beta_{5D} (D_{t} lnP_{t} )$ replace $\beta_{5,t} lnP_{t}$; we therefore have Model (11)

11 $$\begin{aligned} lnC_{it} & = C + \beta_{1,t} lnY_{t} + \beta_{2,t} lnS_{t} + \beta_{3,t} lnE_{t} + \beta_{4,t} lnT_{t} \\ & \quad + \beta_{5A} (A_{t} lnP_{t} ) + \beta_{5D} (D_{t} lnP_{t} ){ + }\mu_{t} \\ \end{aligned}$$

## Results and discussion

The results of Model (11) are presented in Table 6, in which AP and DP represent rising and falling relative energy prices, respectively.

**Table 6:** The regression results regarding the effectiveness of relative prices in rising and falling periods with respect to industrial energy consumption in China

	c	AP	DP	Y	S	E	T	
C_1_	−3.9126 (−2.130**)^†^	−0.1207 (−0.972)	−0.1278 (−0.966)	0.1430 (0.6107)	−0.1421 (−0.441)	3.0930 (8.6167***)	0.3542 (3.0364***)	R^2^ = 0.9960 DW = 1.8515
	–	−0.3520 (−4.334***)	−0.3564 (−3.991***)	0.9063 (10.055***)	1.6272 (10.005***)	–	–	R^2^ = 0.9966 DW = 1.8975
C_2_	−8.5171 (−4.638***)	−0.1207 (−0.972)	−0.1278 (−0.966)	−0.8569 (−3.660***)	−0.1422 (−0.442)	3.0929 (8.6190***)	0.3542 (3.0373***)	R^2^ = 0.9904 DW = 1.8514
	12.2223 (−6.963***)	−0.2353 (−5.545***)	−0.2657 (−6.204***)	–	−0.9136 (−3.381***)	3.9045 (10.8265***)	–	R^2^ = 0.9824 DW = 1.7998

*^,^ **^,^ *** Rejection of the null hypothesis at the 10, 5 and 1 % confidence levels, respectively. For the regression model C_1_, which includes Y, S and P, the iterative estimation method is used because the regression results that use OLS are not sound

^†^The values in parentheses are t statistics

The results reveal that, at present, the output and structure of economic development and the energy consumption structure are key factors affecting total energy consumption, and technological progress and rising relative energy prices can restrain total energy consumption and decrease energy intensity to a certain extent. Moreover, the reactions of both total energy consumption and energy intensity to rising and falling relative energy prices are quite similar. Concretely speaking, the depressing and upward effects of rising and falling relative energy prices on total energy consumption are 0.3520 and 0.3564, respectively; in addition, these effects are 0.2353 and 0.2657 with respect to energy intensity. These results show that the effect of energy prices on energy consumption is symmetric.

## Conclusions

First, under an industry-driven economic growth model, because the demand for energy was rigid and energy prices were not market-oriented, the depressing effect of rising energy prices on total energy consumption does not manifest. However, in certain periods, commodity prices and (therefore) overall price levels are stable, and energy prices enable a degree of regulation of energy consumption through their effects on economic aggregates, the industrial structure and the energy structure, causing the overall effect of energy prices on energy consumption to be negative. This result demonstrates that rising energy prices improve energy efficiency and reduce energy intensity in China to a certain extent, particularly when price levels are relatively stable.

Second, the effects of rising and falling relative prices on energy consumption in China are significantly asymmetric because the depressing effect of rising energy prices on total energy consumption and intensity is far smaller than the upward effect of falling energy prices. This result indicates first that low energy prices resulting from the present mandatory energy-pricing mechanism is the main factor hindering energy-saving and emission reductions; thus, market-oriented reform of energy prices is a vital policy direction for energy conservation; second, this result shows that adjustments to the industrial structure and particularly the internal industrial structure is key to energy conservation. However, a guiding force is necessary to achieve this result. Market-oriented energy prices can effectively guide the optimization and adjustment of the industrial structure. Thus, in practice, more attention should be focused on coordination between adjustments to the industrial structure and the marketization of energy prices.

Third, market-oriented reform is vital to energy savings and emissions reductions in China. To fulfill these goals, government control of energy prices should be reduced gradually, which will lead to a more efficient price-discovery process and result in energy prices being better reflected by supply and demand and resource scarcity. Moreover, pushing to phase out preferential policies for energy enterprises, adjusting energy price subsidies gradually to prevent misleading information regarding energy consumption and guiding enterprises to accelerate technological innovation and improve energy efficiency will result in boosting industrial energy savings and emissions reductions. In particular, energy price reform may reduce energy use (via energy efficiency and restructuring), but it may have an undesired effect on GDP (Aucott and Hall 2014), namely, a decrease in output. Therefore, this approach should be examined in future research, especially an analysis of the relationship between energy saving and GDP declines.

## Abbreviations

SSM: state-space mode
CPI: consumer price index
RPI: retail price index

## Footnotes

1 National Development and Reform Commission of China (NDRC).

2 See the speech by Hu Zucai (Deputy Director of the NDRC of China) at the press conference of the State Council (Oct. 21, 2015).

3 The data embodied in Fig. 1 are subject to limited availability, and the sample period is therefore 1988–2012; in addition, China’s energy prices are replaced by an index of raw materials and fuel purchase prices. The foreign energy price is proxied by the Brent spot crude oil price in RMBs. Both prices use 1988 as their base year.

4 Wei and Lin (2007) and Yang (2009) define relative energy prices at home and abroad as the ratio of the domestic energy price index to the international energy price index.

5 The definition of the real interest rate is the nominal interest rate excluding the inflation factor (inflation rate or CPI growth rate), and the equation is $Real\,{\kern 1pt} interest\,rate = Nominal\,interest\,rate - inflation\,rate$. Moreover, the real product price index is the nominal price excluding the inflation factor, and the general equation is $Real\,{\kern 1pt} produce\,index = nominal\,price \times \frac{{Current\,{\kern 1pt} CPI}}{Base\,CPI}$. Additionally, Yang (2009) defines the real energy price as the nominal price divided by a GDP deflator.

6 Because the main source of CO_2_ emissions is energy consumption, as mentioned previously, we use the equation directly to analyze energy consumption.

7 This paper focuses on a general condition of economic development, so we use GDP instead of GDP capita.

8 There is no uniform energy price index for China (or industrial energy price index), so some studies use an index of raw materials and the fuel purchasing price, while others use the weighted average price of different varieties of energy to represent the domestic energy price. We use the former in this paper. Moreover, the general price level is usually represented by the consumer price index (CPI) and the retail price index (RPI). The CPI is a consumptive index, whereas the RPI is a productive index. Because the energy consumption we focus on in this paper is also a productive variable, RPI is a more reasonable measure to represent the price level that is influenced by productive variables in practice.

9 Data are taken from the “China Statistical Yearbook,” the “China Energy Statistical Yearbook” and the INFOBANK databases.

10 To clarify the effect of relative energy prices P, we also estimate the model without P, and we find that both the estimations of Y and S are not significant in the model that uses C_1_ as the dependent variable (P values are 0.4866 and 0.3948, respectively). Moreover, the effect of T on C_1_ is positive, and this result is inconsistent with the theory. In general, the estimation of the model with P is better than that without P, which mainly reflects three aspects: first, for the model with P, the pulling effect of the aggregate output Y on energy consumption decreases to some extent; second, the depressing effects of the industrial structure S and the energy structure E on energy consumption increases; and third, the variable P significantly passes the related statistical tests. These results not only highlight the reasonableness of Model (7) but also provide a good basis for analyzing the regulatory effects further.
